# Intracellular PD Modelling (*PD*_i_) for the Prediction of Clinical Activity of Increased Rifampicin Dosing

**DOI:** 10.3390/pharmaceutics11060278

**Published:** 2019-06-13

**Authors:** Ghaith Aljayyoussi, Samantha Donnellan, Stephen A. Ward, Giancarlo A. Biagini

**Affiliations:** Centre for Drugs & Diagnostics, Liverpool School of Tropical Medicine, Pembroke Place, Liverpool L3 5QA, UK; ghaith@lstmed.ac.uk (G.A.); Samantha.Donnellan@lstmed.ac.uk (S.D.); steve.ward@lstmed.ac.uk (S.A.W.)

**Keywords:** pharmacokinetic/pharmacodynamic modelling, rifampicin, high dose, tuberculosis, infectious diseases, *Mycobacterium tuberculosis*

## Abstract

Increasing rifampicin (RIF) dosages could significantly reduce tuberculosis (TB) treatment durations. Understanding the pharmacokinetic-pharmacodynamics (PK–PD) of increasing RIF dosages could inform clinical regimen selection. We used intracellular PD modelling (*PD*_i_) to predict clinical outcomes, primarily time to culture conversion, of increasing RIF dosages. *PD*_i_ modelling utilizes in vitro-derived measurements of intracellular (macrophage) and extracellular *Mycobacterium tuberculosis* sterilization rates to predict the clinical outcomes of RIF at increasing doses. We evaluated *PD*_i_ simulations against recent clinical data from a high dose (35 mg/kg per day) RIF phase II clinical trial. *PD*_i_-based simulations closely predicted the observed time-to-patient culture conversion status at eight weeks (hazard ratio: 2.04 (predicted) vs. 2.06 (observed)) for high dose RIF-based treatments. However, *PD*_i_ modelling was less predictive of culture conversion status at 26 weeks for high-dosage RIF (99% predicted vs. 81% observed). *PD*_i_-based simulations indicate that increasing RIF beyond 35 mg/kg/day is unlikely to significantly improve culture conversion rates, however, improvements to other clinical outcomes (e.g., relapse rates) cannot be ruled out. This study supports the value of translational *PD*_i_-based modelling in predicting culture conversion rates for antitubercular therapies and highlights the potential value of this platform for the improved design of future clinical trials.

## 1. Introduction

Tuberculosis, caused by *Mycobacterium tuberculosis (Mtb)*, remains the leading cause of death from a single infectious agent [1]. With an estimated 4000 deaths per day, and 10 million newly infected people each year, the global health community have pledged to lead the fight against this disease [2]. Treatment takes a minimum of six months of multi-drug therapy with rifampicin (RIF) being the key drug responsible for shortening treatment down from 18 to six months [3,4] when administered at 600 mg daily (10 mg/kg). At this standard daily dose, RIF has been well tolerated; however, dosing of RIF is primarily based on cost constraints rather than vigorous toxicity testing [5], and many studies have highlighted the advantages of increasing this recommended prescription. For example, in vitro and in vivo (animal) studies have evidenced faster bacillary clearance rates, lower disease relapse rates, decreased resistant strain emergence and evidence of shortening the treatment period when RIF levels were increased [4,6,7,8]. 

Previous pharmacokinetic-pharmacodynamic (PK–PD) analyses have highlighted the potential for accelerating TB treatment through higher doses of RIF [9]. It has frequently been revealed in clinical studies than increasing RIF dosage results in favorable bacteriological findings [5,7,10,11]. This has led to repeated calls for changing the clinical practice by increasing the standard dose of RIF from 10 mg/kg [12,13]. However, only one controlled study to date has systematically studied the effects of increasing the dosage of RIF up to 35 mg/kg with a full follow-up of patients receiving standard or elevated RIF treatment [14]. This work was further supported by post-modelling analyses that related drug exposure to treatment response [15]. The work described herein uses the results from this clinical trial to compare and validate our mathematical predictions.

We previously designed a model using an imaging-based approach to define the killing kinetics of anti-TB drugs inside macrophage cells [16], and showed that intracellular *Mtb* growth and kill dynamics (*PD*_i_) can be used to predict the bacteriological outcomes in patients receiving treatment. In this work, we demonstrate how *PD*_i_-based modelling can be used to predict the benefits of increasing RIF dosages from 10 mg/kg up to 35 mg/kg, and we validate our *PD*_i_ modelling simulations by comparing our clinical predictions (based on in-vitro intracellular time kill data) to the observed clinical findings reported in Boeree et al. [14]. *PD*_i_ modelling is a deterministic PK–PD mathematical model, based on *in vitro Mtb* growth and kill rates in both intracellular and extracellular environments, which, when linked to the known PK parameters of each drug, can predict the speed of clinical response to treatment.

## 2. Materials and Methods

### 2.1. PD*_i_* Modelling

**Figure 1:**
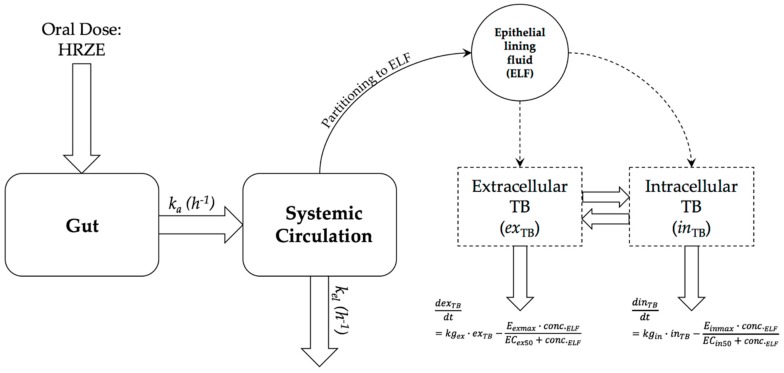
Mathematical model used to predict overall *Mtb* count in silico. *k_a_* is rate of absorption in hours, *k_el_* is the rate of elimination from the systemic circulation per hour. ELF concentration is assumed to be a fraction of the systemic circulation concentration of each drug at any given time. Respectively, ex_TB_ and in_TB_ are the number of extracellular and intracellular tuberculosis bacteria, kg_ex_ and kg_in_ the rates of extracellular and intracellular bacterial growth, E_exmax_ and E_inmax_ are the maximal kill rates against extracellular and intracellular bacteria, and EC_50ex_ and EC_50in_ are the half-maximal-effect drug concentrations in the extracellular or intracellular environment for each drug. conc._ELF_ is the drug concentration in the ELF.

*PD*_i_ modelling was performed using Pmetrics [17]. We used extracellular and intracellular kill rates of TB drugs (previously reported in Aljayyoussi et al. [16]) to build a predictive PK–PD model for the prediction of the efficacy of isoniazid (H), rifampicin (R or RIF), pyrazinamide (Z), and ethambutol (E)—a combination commonly abbreviated as HRZE (Figure 1).

Drug PKs are described according to a one-compartment oral absorption model used to predict plasma drug concentration for each drug detailed in Equations (1)–(4), where *Gutx* is a drug mass in the gut compartment at any given time, *Plasmax* is the total drug mass in plasma at any given time, and *k_a_* and *k*_e_ are the rates of absorption and elimination in hours, respectively. *V/F* is the reported volume of distribution of each drug, ELF/Plasma Ratio is the constant ratio between the epithelial lining fluid (ELF) and plasma, as reported in the literature for each drug. ELF drug concentration is assumed to be equal to a constant fraction of plasma drug concentration at any given dosage. The fraction for each drug is taken from the literature, where plasma and ELF AUC are simultaneously reported [18,19,20,21,22].

Importantly, for the work reported herein, the PK parameters that define plasma exposure to each drug (namely, *k**_e_*** and *V/F*) were obtained from the same clinical study to which we compare our predictions [14], where RIF has different PK parameters at different RIF dosing, leading to a non-linear relationship between increase of dose and increase of plasma exposure. Although V/F and CL/F values for RIF are reduced as dosage is increased, it is unlikely that RIF PK follows non-linear PK properties. That is because the overall elimination half-life remains largely unchanged as the dose increases from 10 to 20 mg/kg, and then to 35 mg/kg. The improvement in PK properties as the dose is increased is hence more likely related to an improvement in bioavailability. We therefore modeled each RIF dose independently with a separate set of PK parameters rather than using a Michaelis–Menten model.

The ELF drug concentration is then assumed to drive overall drug activity for the PD component of the model. *Mtb* is assumed to exist both extracellularly and intracellularly with a ratio of 95:5 [16]. Drug dynamics are different for each of these populations with the different maximal kill rates (*E_max_*) and half-maximal concentration (*EC_50_*) values reported previously [16]. An *E_max_* model is assumed to be the driver of the PK–PD relationship for all four drugs. The PD component of the model is described by Equations (5) and (6), where, respectively, *ex_TB_* and *in_TB_* are the number of extracellular and intracellular bacilli, *kg_ex_* and *kg_in_* the rates of extracellular and intracellular bacterial growth, *E_exmax_* and *E_inmax_* are the maximal kill rates against extracellular and intracellular bacilli, and *EC_50ex_* and *EC_50in_* are the half-maximal-effect drug concentrations in the extracellular or intracellular environment for each drug. *conc._ELF_* is the drug concentration in the ELF. The interchange between extracellular and intracellular TB is unknown and was lumped within the overall intracellular and extracellular kill rates.

For drug combinations, an algorithm is used where the overall kill rate at any given time is equal to the kill rate of the drug with the highest kill rate at that given time point (this kill rate is dependent on the changing of a drug’s concentration as well as its constant PD parameters *(**EC_50_*** and ***E_max_***) at any given time point). The model therefore assumes that there are no positive or negative PD interactions between the drugs.

(1) $$\frac{dGutx}{dt}=-Gutx\cdot k_{a}$$

(2) $$\frac{dPlasmax}{dt}=Gut\cdot k_{a}-Plasmax\cdot k_{el}$$

(3) $$Plasma\;conc.\;(ng/mL)=\frac{Plasmax}{V/F}$$

(4) ELF conc. (ng/mL)=Plasma conc.(PlasmaELF) Ratio

(5) $$\frac{dex_{TB}}{dt}=kg_{ex}\cdot ex_{TB}-\frac{E_{exmax}\cdot conc._{ELF}}{EC_{ex50}+conc._{ELF}}$$

(6) $$\frac{din_{TB}}{dt}=kg_{in}\cdot in_{TB}-\frac{E_{inmax}\cdot conc._{ELF}}{EC_{in50}+conc._{ELF}}$$

The total number of mycobacteria are assumed to be equal to the sum of extracellular and intracellular mycobacteria at any given time. We assumed an initial burden of 10^7^ CFUs in all patients with a range of 10^5^–10^8^; this value is derived from data reported by de Knegt et al. [23]. Previous sensitivity analysis showed that the ratio of intracellular to extracellular TB burden plays little role in overall prediction outcomes over a range of 5%:95% to 95%:5%, intracellular:extracellular [16].

For Monte Carlo simulations, we used previous reports to estimate variability of PK parameters [24,25,26] and follow a simple log-normal distribution around the geometric mean value reported in Boeree et al. for each drug (Table S1). The number of simulations generated for each scenario was made to equal the number of subjects in each arm of the Boeree et al. study to allow for like-to-like comparisons between *PD*_i_ model predictions and observed clinical outcomes. The simulations were performed using the simulator tool in Pmetrics [17] at a differential step of 24 h for a total of 4320 h (six months) for each treatment arm.

We performed overall sensitivity analysis using the flexible modelling environment (FME for inverse modelling, sensitivity, identifiability and Monte Carlo analyses; authors: Karline Soetaerrt and Thomas Petzoldt) with R version 3.4.2 [27]. We report the *l*^1^ norm and *l*^2^ norm values, which are a measure of how sensitive the model is to small changes in each parameter used in the simulation. The higher these values are for a parameter, the more sensitive the model to the parameter.

Additionally, we performed *PD*_i_ modelling using an auto-induction, non-linear pharmacokinetic model for rifampicin, as reported in Svensson et al. [28] for the PK arm of the model, and we report results using it separately in the Supplementary Information part of the paper.

### 2.2. Hazard Ratio Calculation

Hazard ratios (HRs) were calculated using the cox regression survival tool in IBM SPSS Statistics version 24.0 [29]. Survival data were generated at a step of 1 week for a total of 8 or 12 weeks from each *PD*_i_ modelling simulation. The Monte Carlo simulation output from Pmetrics was converted using R version 3.4.2 [27] to survival data (i.e., Kaplan–Meier style data). HR values were calculated between standard HRZE therapy and the intervention arm RIF_35_HZE (same as HRZE but with a 35 mg/kg dose of RIF for the first 12 weeks) or RIF_70_HZE, same as HRZE but with a hypothetical 70 mg/kg dose of rifampicin for the first 12 weeks. HR were generated as an Exp(B) value in SPSS with 95 confidence intervals (CIs). HRs were also similarly calculated for simulating increasing RIF exposures from 15 mg/kg (RIF_15_HZE) up to 60 mg/kg (RIF_60_HZE). In hazard ratio calculations, it was assumed that each simulated patient would have a negative culture conversion status if the total burden was <1 CFU/mL. The previous value is based upon the sensitivity threshold of the mycobacteria growth indicator tube (MGIT) technique [30].

### 2.3. PK Monte Carlo Simulations

PK simulations where performed similarly using the Pmetrics simulator tool at 0.1 h intervals for 24 h at steady state applying the same PK parameters reported in Boeree et al. (2017). For the 70 mg/kg dose, the parameters of 35 mg/kg dose were used. Exposure at seven to eight days post-treatment initiation was plotted to represent steady-state PK exposure of RIF. ELF exposure was calculated by multiplying plasma values at each time point by the appropriate ELF penetration ratio as reported for each drug in the review by Keim et al. [20]. The PK element of the *PD*_i_ modelling was performed similarly.

## 3. Results

### 3.1. PD*_i_* Modelling Predicts Efficacy of Standard and High-Dose RIF-Containing Treatments

As shown in Table 1, *PD*_i_ modelling predicts 94% of patients achieve MGIT negative culture conversion by 26 weeks with the standard HRZE treatment (10 mg/kg RIF, 5 mg/kg isoniazid, 25 mg/kg pyrazinamide, and 15–20 mg/kg ethambutol for 8 weeks followed by 18 weeks of standard dose Rif and isoniazid), which is comparable to an observed 82% of patients achieving the same result in the Boeree et al. clinical trial [14]. We predict a median of eight weeks to achieve culture conversion compared to an observed nine weeks in the same clinical trial (Table 1).

However, the model predicted that nearly all patients (99%) would achieve culture conversion by 26 weeks with RIF_35_HZE treatment, whereas patients receiving this treatment had no overall improvement in achieving culture conversion at 26 weeks (only 81% of patients achieved culture conversion in RIF_35_HZE, compared with 82% in the standard treatment). Additionally, comparing RIF_35_HZE and HRZE, we predict an HR of 2.04 (1.41–2.94) at eight weeks, which is comparable to the observed adjusted HR of 2.06 (1.26–3.38). Similarly, we predict an HR of 1.68 (1.21–2.32) at 12 weeks, comparable to the observed HR of 1.78 (Table 1).

**Figure 2:**
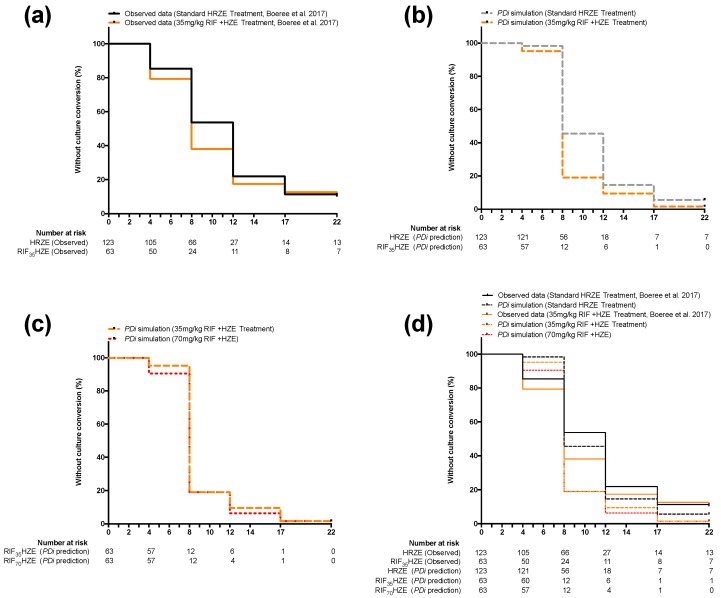
Simplified Kaplan–Myer curves comparing observed and predicted clinical outcomes (defined as conversion to culture negative status using MGIT over time) of the standard RIF dose-containing regimen (HRZE) versus the 35 mg/kg RIF dose-containing regimen (H_35_RZE): (**a**) reported data in Boeree et al. comparing HRZE (solid black line) and H_35_RZE (solid orange line); (**b**) mathematically predicted outcome (using *PD*_i_ modelling) for HRZE (dashed black line) and H_35_RZE (dashed orange line) applying the same conditions, patient numbers and PK parameters reported in the Boeree et al. clinical trial; (**c**) mathematically generated comparison between H_35_RZE (orange dashed line) and a 70 mg/kg RIF dose-containing regimen (H_70_RZE) (red dotted line); (**d**) overlaid Kaplan–Myer curves seen in panels (**a**), (**b**) and (**c**).

Figure 2 shows a comparison between simplified Kaplan–Meier curves generated from *PD*_i_ simulations and ones that were adapted from data reported in Boeree et al. [14]. For simplification, and because they were the data explicitly reported in the clinical study, the data shown in the figure are for 4, 8 12, 17, and 22 weeks only. *PD*_i_ simulation HR values, however, were calculated at a 1-week step for 8 or 12 weeks, as described in the methods.

Using the Svensson et al. [28] holistic PK model instead of the individual PK parameters for the subjects of each arm in the study as reported in Boeree et al. [14] resulted in a similar outcome. The model still predicted a significant improvement in culture negative conversion at 8 and 12 weeks (HR = 1.53, *p* < 0.01, and 1.33, *p* < 0.01, respectively) and a minimal improvement when dose was increased beyond 35 mg/kg (Table S2, Figure S1).

### 3.2. PD*_i_* Modelling Suggests No Further Reduction in Time to Culture Conversion Rates by Increasing RIF Dose Beyond 35 mg/kg

To test whether increasing dosage beyond 35 mg/kg would lead to any benefit in accelerating treatment outcome, a hypothetical 70 mg/kg RIF-containing regimen (RIF_70_HZE) was modeled and compared to the standard treatment. Figure 2, (**c**) shows that increasing the dose to 70 mg/kg would result in a negligible improvement in time-to culture conversion. Indeed, the predicted HR at eight weeks from RIF_70_HZE (2.16) offers a limited improvement to the predicted HR for RIF_35_HZE at the same time point (2.04) when compared to control treatment (HRZE) (see Table 1). The HR between RIF_35_HZE and RIF_70_HZE is statistically insignificant (*p* > 0.05, assuming 123 patients in each arm) at 1.12 (0.81–1.59).

### 3.3. PD*_i_* Modelling Predicts a Dose-Effect Relationship of RIF

**Figure 3:**
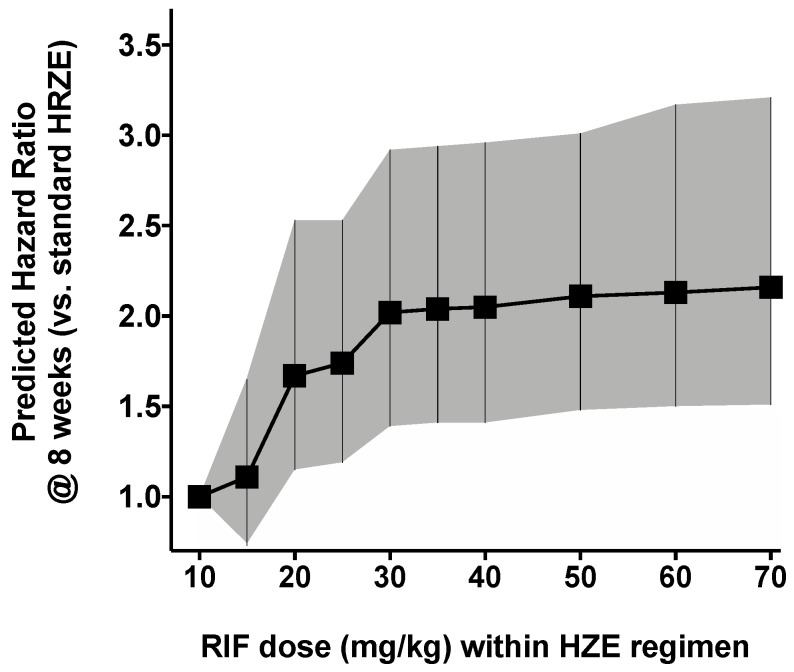
Predicted hazard ratio for different RIF dosing levels: 10 mg/kg up to 70 mg/kg (RIF_70_HZE) compared with the standard HRZE treatment. The hazard ratio was calculated over a period of eight weeks for each dosing level. Black squares are calculated hazard ratios and the grey shaded area is the confidence interval.

We simulated hypothetical doses from 10 mg/kg up to 35 mg/kg to assess the effect of increasing doses of RIF in the treatment regimen upon the overall effect. This was done assuming different PK parameters for each RIF dosing band, as previously described. Simulations show an increase in eight-week HR is achieved as the RIF dose increases from 10 mg/kg (HRZE) up to 15 mg/kg (RIF_15_HZE) (HR = 1.11 (0.74–1.65), *p* < 0.01), 20 mg/kg (RIF_20_HZE) (HR = 1.68 (1.15–2.52), *p* < 0.001), 25 mg/kg (RIF_25_HZE) (HR = 1.74 (1.19–2.55), *p* < 0.001) and 30 mg/kg (RIF_30_HZE) (HR = 2.02 (1.41–2.92), *p* < 0.001). The RIF_30_HZE result is of particular importance as it shows very little difference from the RIF_35_HZE, suggesting the need to probe the additional benefits higher doses of RIF provide to patients of in future studies. The increase of HR with increasing RIF dosage is further visualized in Figure 3.

Significantly, *PD*_i_ predicts that replacing EMB with a slightly higher RIF dose (20 mg/kg) (RIF_20_HZ) results in no improvement in treatment outcome (time-to culture conversion), where HR is insignificant when compared with the standard HRZE treatment (HR = 0.98 (0.69–1.39), *p* = 0.721). This compares with an insignificant HR value in Boeree et al., where a dose combination of RIF (20mg/kg), INH, PZA, and a new drug, SQ109, were compared to HRZE (adjusted HR = 1.06 (0.74–1.52), *p* = 0.75).

### 3.4. PK Simulation Analysis

**Figure 4:**
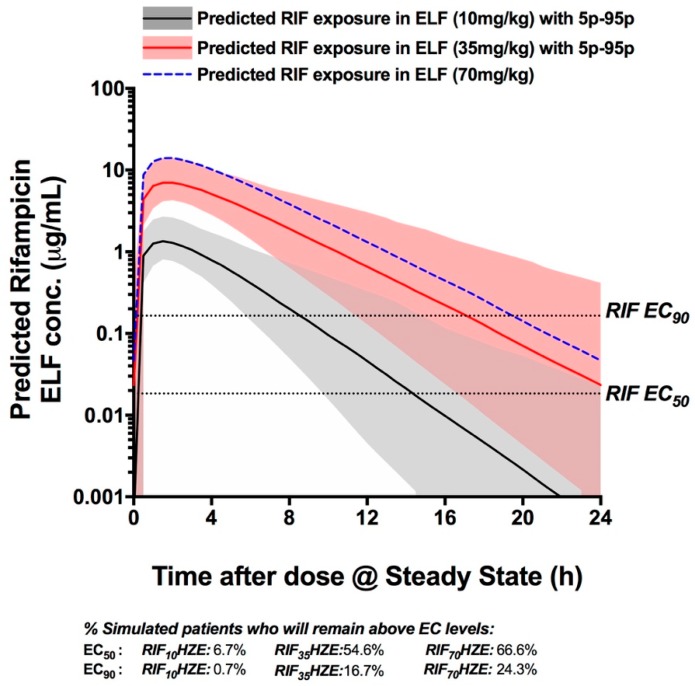
Monte Carlo PK simulations based on the parameters reported in Boeree et al. The simulations show the median exposures (lines) and 9–95 percentile range (shaded areas) for each treatment arm (Standard HRZE (R_10_HZE), solid black line and grey shading. Treatment containing 35 mg/kg RIF with HZE (RIF_35_HZE) is represented by a solid red line and orange shading; a hypothetical 70 mg/kg RIF with HZE that assumes similar PK parameters to RIF_35_ (RIF_70_HZE) is represented by a dotted blue line; percentile range not shown for ease of overall comparison.

Monte Carlo simulations of the PK exposure were performed to predict the ELF exposure to RIF at doses of 10, 35, and 70 mg/kg. Out of the 300 subjects simulated in the Monte Carlo simulations, we identified the proportion of patients who would remain above the intracellular EC_50_ (18.4 ng/mL) and EC_90_ (165.1 ng/mL) levels for the whole duration of treatment. The analysis shows that very few patients would maintain ELF exposure of RIF above the EC_50_ when they take a standard 10 mg/kg RIF dose (6.7% of patients over 24 h), while over 54% of patients would remain above this level for the whole duration of treatment when receiving RIF_35_HZE. The proportion of patients with RIF ELF exposure above the EC_50_ for the whole duration only increases by 12% with a hypothetical dose of 70 mg/kg. A similar picture can be drawn when analyzing the proportion of patients whose RIF ELF levels will remain above the EC_90_ cut-off, as can be seen in Figure 4 (Figure S2 contains the plasma PK simulations assuming the same conditions). Importantly, the analysis shows that the clear majority of patients in the RIF_35_HZE arm (97%) would remain above the EC_50_ level of RIF for at least 60% of the treatment duration time, while a minority of patients (33.4%) would achieve that effect when they receive a standard HRZE treatment. Figure S2 represents the predicted plasma exposure to RIF at the standard, 35 mg/kg, and a hypothetical 70 mg/kg dose of RIF.

## 4. Discussion

RIF dosing was primarily standardized based on cost; however, the drug is now widely available with little financial burden [31]. Being readily available throughout the world at a low cost with a good safety profile for doses up to 40 mg/kg [28] means that increasing standard RIF dosing could be easily and rapidly applied, and therefore should be seriously considered in future planning for treatment regimens.

The *PD*_i_-based PK–PD modelling and simulations provide the means to predict the efficacy of the standard first-line TB treatment at the level of time-to culture-conversion. *PD*_i_-based PK–PD modelling can also be utilized to predict the superiority of increasing RIF dosing in patients between 8 and 12 weeks of treatment, whereby our predicted HRs are comparable to those reported from the clinic, thus giving us confidence in our model at the initial stage of treatment. Our model suggests that increasing dosage of RIF results in an improved bacteriological outcome until the dose reaches 30–35 mg/kg, beyond which a great improvement in patient outcome, in terms of bacillary culture conversion rates, is not evidenced. For example, RIF_30_HZE, RIF_35_HZE and RIF_70_HZE have similar predicted HRs that are not statistically different from each other, suggesting that increasing the dose beyond 30–35 mg/kg might not be any more useful when comparing HRs at eight weeks. This implicates doses beyond 70 mg/kg and, despite non-linear pharmacokinetic properties, it is expected that the rate of bacillary clearance will plateau after 35 mg/kg dosing. It is important to underline, however, that there may be significant additional patient benefits derived from >35 mg/kg RIF doses that are not captured here using the *PD*_i_-based PK–PD modelling e.g., relapse rates.

A further observation derived from comparing the *PD*_i_-based simulations with clinical data is that there is a sub-population of patients (~20%) who do not benefit from an increased dose of RIF (even at 26 weeks post-treatment), whereas our modelling predicts that increasing the RIF dose for the first 12 weeks of treatment should ultimately increase the number of patients who achieve culture conversion at 26 weeks. This discrepancy is reflected by the fact that *PD*_i_ modelling can actually predict the median time and 25th quartile (Q_25_) to achieve culture conversion in the RIF_35_HZE arm (median: 40 days predicted vs. 48 days observed; Q25: 34 days predicted vs. 34 days observed), but when comparing the 75th quartile (Q_75_), the model fails to predict patient response (51 days predicted vs. 69 days observed). This contrasts with our prediction of the standard treatment where lower and higher quartiles are well matched when comparing predictions and observations.

We simulated the same (limited) number of patients that were used in the comparator clinical trial to display how results from *PD*_i_ modelling can be compared head to head with field results. However, simulations using a higher number of patients results in similar outcomes. For example, when increasing the sample size from 123 and 63 patients in the control and high RIF groups, respectively, to 4000 patients in each arm the HR remains similar, albeit with a narrower CI interval (HR = 2.01 [1.96–2.10]).

This discrepancy between our prediction of culture conversion at 26 weeks versus observed data is important, as it highlights the importance of investigating the uniqueness of patients who respond poorly to increased doses of RIF. This could potentially be due to the presence of a subpopulation of *Mtb* that responds slower to RIF in those particular patients, or it could be due to higher disease burden/more severe pathology. Another potential reason is that the Boeree et al. clinical trial had a limited number of patients in the high dose RIF group (63 patients) to discern a difference at the 26-week point of the study.

While our *PD*_i_ modelling does not predict a significant improvement beyond the 35 mg/kg RIF level, it is important to explore further how higher doses might change clinical outcomes in clinical trials. For instance, doses beyond 35 mg/kg might help the sub-population of patients who did not respond at the 35 mg/kg mark, and which our model has ultimately failed to predict. However, from a mathematical modelling point of view, any increase in dosage beyond 35 mg/kg is predicted to result in no significant improvement in the overall clinically-observable (sputum sterilization) rate of bacillary clearance.

Previous PK-PD modelling used to predict the activity of RIF based on hollow fiber models has been reported [32]. These have predicted significant improvements as RIF doses are increased from 10 mg/kg to 35 mg/kg, which matches our predictions but also predicts a potentially modest improvement in overall activity when dosage is increased to 50 mg/kg. We predicted no more than a 5% improvement when increasing dosage from 35 to 50 mg/kg. This remains to be investigated in future clinical trials.

Our modelling also shows that INH and PZA play a negligible role in the overall outcome of treatment containing RIF, EMB, INH, and PZA. The activity is mainly driven through RIF and then by EMB, while INH and PZA had next to no effect on the overall outcome (Table S2). This agrees with previous modelling that indicated limited effects of the two drugs [33]. Again, it is important to underline however, that there may be significant additional patient benefits derived from inclusion of INH and PZA within the drug combination that are not captured here using the *PD*_i_-based PK–PD modelling e.g., relapse rates and generation of drug resistance.

Using ELF PK analysis, we provide further evidence of why increasing RIF dosage from 10 to 35 mg/kg might result in a significant improvement of clinical outcomes, whilst increasing the dosage further might not be as beneficial. While increasing the dosage from 10 to 35 mg/kg will increase the number of patients who sustain ELF levels that are continuously above the EC_50_ by 50%, a further increase to 70 mg/kg only results in an extra 12% increase in patients sustaining such levels. This is partly due to the non-linear relationship between RIF doses and systemic exposure, where a 3.5-fold increase in dosage leads to up to a sevenfold increase in exposure. The non-linear relationship between RIF dosage and its exposure has been previously reported, including cases where the dose is increased by 1.5–2 fold [34,35].

Our study’s limitations highlight some important deficiencies in available data that would be important to fulfill in the future. We still have a poor understanding of ELF exposure to RIF at elevated doses. Our current assumption is that the plasma–ELF ratio remains constant over increasing doses, which might not necessarily be true. This deficiency needs to be addressed by studies that quantify RIF ELF levels at higher doses in vivo and clinically. The discrepancy between the end result of our modelling highlights the possible complexity of the PK–PD relationship for RIF’s increasing doses, and how conventional PK–PD analyses might fall short of explaining rather complex dynamics that change over time, as well as the possibility of having numerous sub-bacterial populations that respond to chemotherapy in unique ways. Efforts are already taking place to improve translational modelling activities and reduce such gaps. Translational modelling has also been improved recently [33] to further bridge the gap between animal models and clinical trials, where a large gap exists between the two that could be attributed to a large number of factors including disease pathology (e.g., cavitary and non-cavitary disease) as well as natural immunological responses in the host against TB.

## Figures and Tables

**Table 1:** Comparison between observed clinical outcomes (based on MGIT culture conversion results) in Boeree et al. (HRZE vs. RIF_35_HZE) and mathematically simulated outcomes using *PD*_i_ modelling. The last column displays the results for a simulation of a hypothetical 70 mg/kg RIF dose-containing regimen (RIF_70_HZE) using *PD*_i_ modelling. Hazard ratios are comparisons to the control treatment within the observation or simulation groups.

	Boeree et al. (2017) [14] (Observed)		*PD*_i_ Prediction		
	Standard HRZE	H_35_RZE	Standard HRZE	H_35_RZE	H_70_RZE
**Total in Analysis**	123	63	123	63	63
Hazard ratio over 8 weeks (CI) *	N/A	1.73 (1.07–2.82), *p* = 0.004 (unadjusted)			
	N/A	2.06 (1.26–3.38), *p* = 0.004 (adjusted)	N/A	2.04 (1.41–2.94), *p* < 0.001	2.16 (1.50–3.12), *p* < 0.001
Hazard ratio over 12 weeks (CI)	N/A	1.46 (1.02–2.11), *p* = 0.04 (unadjusted)			
	N/A	1.78 (1.22–2.58), *p* = 0.003 (adjusted)	N/A	1.68 (1.21–2.32), *p* < 0.001	1.86 (1.35–2.57), *p* < 0.001
No. of culture conversions during 26-weeks (MGIT **) (% of patients)	101 (82%)	51 (81%)	104 (94%)	62 (99%)	63 (100%)
Median time to culture conversion (IQR) ***	62 (4–83)	48 (34–69)	55 (41–76)	40 (34–51)	39 (32–48)

* CI: Confidence Interval; ** MGIT: Mycobacteria growth indicator tube; *** IQR: Interquartile range.

## Supplementary Materials

The following are available online at https://www.mdpi.com/1999-4923/11/6/278/s1. Table S1 Parameters used in PDi simulations for each TB drug; Figure S1 Predictions of PDi modelling when using the auto-induction non-linear PK model reported by Svensson et al.; Table S2 Comparison between observed clinical outcomes (based on MGIT culture conversion results) in Boeree et al. (2017) (HRZE VS. RIF35HZE) and mathematically simulated outcomes using PDi modelling but using the Svensson et al. [28] non-linear PK model for Rifampicin. The last column displays the results for a simulation of a hypothetical 70 mg/kg RIF dose containing regimen (RIF70HZE) using PDi modelling. Hazard ratios are comparisons to control treatment within the observation or simulation groups; Table S3 Sensitivity analysis results for the model. L1 and l2 norm values represent the weight of each parameter upon the outcome of the simulation; Figure S2 RIF predicted plasma exposure at different doses overlaid with RIF EC50 and EC90.
