# Supplementary material for: Intracellular PD Modelling (PDi) for the Prediction of Clinical Activity of Increased Rifampicin Dosing
Source: Pharmaceutics. 2019 Jun 13;11(6):278. doi: 10.3390/pharmaceutics11060278 (PMC6630509; doi:10.3390/pharmaceutics11060278)
Supplement: Supplementary file 1 [file pharmaceutics-11-00278-s001.pdf]

pharmaceutics

MDPI

Communication

Intracellular PD Modelling (PDi) for the Prediction of Clinical Activity of Increased Rifampicin Dosing

Ghaith Aljayyoussi†, Samantha Donnellan†, Stephen A. Ward and Giancarlo A. Biagini *

Centre for Drugs & Diagnostics, Liverpool School of Tropical Medicine, Pembroke Place, Liverpool L3 5QA, UK; Samantha.Donnellan@lstmed.ac.uk (S.D.); steve.ward@lstmed.ac.uk (S.A.W.); giancarlo.biagini@lstmed.ac.uk (G.A.B)

* Correspondence: Giancarlo.biagini@lstmed.ac.uk

† These authors contributed equally to this manuscript

Received: 23 April 2019; Accepted: 5 June 2019; Published: 13 June 2019

Supplementary Tables and Figures

Table 1. Parameters used in PDi simulations for each TB drug.

Treatment	Parameter	Intracellular	Extracellular
Control	Kg_{max} (h^{-1} (D.Time)	0.033 (21.0 h)	0.0769–0.045 (9.0 h)
Rifampicin	E_{max} (h^{-1})	0.055	0.178
	EC_{50} (ng/mL)	18.4	5.60
	V/F (L/kg)		
	10mg/kg	1.0	
	20mg/kg	0.87	
	35mg/kg	0.75	
	CL/F (L/h/kg)		
	10mg/kg	0.41	
	20mg/kg	0.29	
	35mg/kg	0.21	
	Plasma:ELF ratio	0.26	
Ethambutol	E_{max} (h^{-1})	0.053	0.142
	EC_{50} (ng/mL)	79.5	264
	V/F (L/kg)	10.3	
	CL/F (L/h/kg)	0.78	
	Plasma:ELF ratio	1.03	
Isoniazid	E_{max} (h^{-1})	0.041	0.710, 0.055
	EC_{50} (ng/mL)	32.1	790
	V/F (L/kg)	2.1	
	CL/F (L/h/kg)	0.45	
	Plasma:ELF ratio	3.53	
Pyrazinamide	E_{max} (h^{-1})	0.043	NA
	EC_{50} (ng/mL)	45.5	NA
	V/F (L/kg)	0.71	
	CL/F (L/h/kg)	0.070	
	Plasma:ELF ratio	19.2	

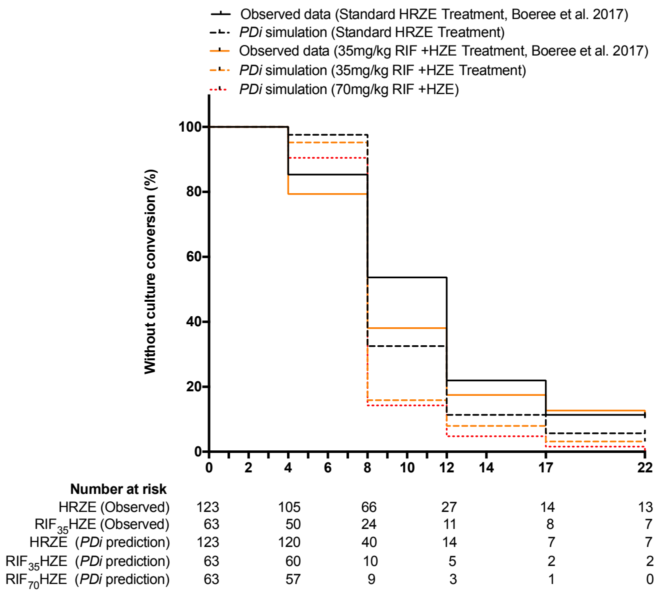

Figure 1. Predictions of PDi modelling when using the auto-induction non-linear PK model reported by Svensson et al. [28].

Table S1. Comparison between observed clinical outcomes (based on MGIT culture conversion results) in Boeree et al. (2017) (HRZE VS. $RIF_{35}HZE$) and mathematically simulated outcomes using PDi modelling but using the Svensson et al. [28] non-linear PK model for Rifampicin. The last column displays the results for a simulation of a hypothetical 70 mg/kg RIF dose containing regimen (**$RIF_{70}HZE$**) using PDi modelling. Hazard ratios are comparisons to control treatment within the observation or simulation groups.

.	**Boeree et al. (2017)—Observed**		**PDi Prediction (Based on Svensson et al. [28] auto-induction non-linear PK model)**		
	**Standard HRZE**	**$H_{35}RZE$**	**Standard HRZE**	**$H_{35}RZE$**	**$H_{70}RZE$**
**Total in analysis**	123	63	123	63	63
**Hazard ratio over 8 weeks [CI]***	N/A	1.73 [1.07-2.82] p=0.004 (unadjusted)			
	N/A	2.06 [1.26-3.38] p=0.004 (adjusted)	N/A	1.52 [1.07-2.15] p<0.001	1.76 [1.25-2.49] p<0.001
**Hazard ratio over 12 weeks [CI]**	N/A	1.46 [1.02-2.11] p=0.04 (unadjusted)			
	N/A	1.78 [1.22-2.58] p=0.003 (adjusted)	N/A	1.33 (0.96-1.83) p<0.001	1.60 [1.16-2.20] p<0.001

Table 3. Sensitivity analysis results for the model. L1 and l2 norm values represent the weight of each parameter upon the outcome of the simulation.

	value	*l*²*-norm*	*l*²*-norm*
E_{max} (RIF)	0.055	1188459.0	517335.7
Initial intracellular bacillary load	5.00E+06	282452.2	124887.9
K_e (RIF)	0.185	282447.9	123397.69
EC_{50} (RIF)	18.4	94266.4	41145.97
V/F (RIF)	60	94132.4	41108.17
K_a (RIF)	0.92	20720.0	9058.57
K_a (EMB)	2.3	222.5	49.2
V/F (EMB)	135	177.4	84.6
EC_{50} (EMB)	168	172.1	82.1
Initial extracellular bacillary burden	9.50E+07	69.5	22.0
K_e (EMB)	0.31	66.9	19.3
E_{max} (EMB)	0.053	15.9	6.3

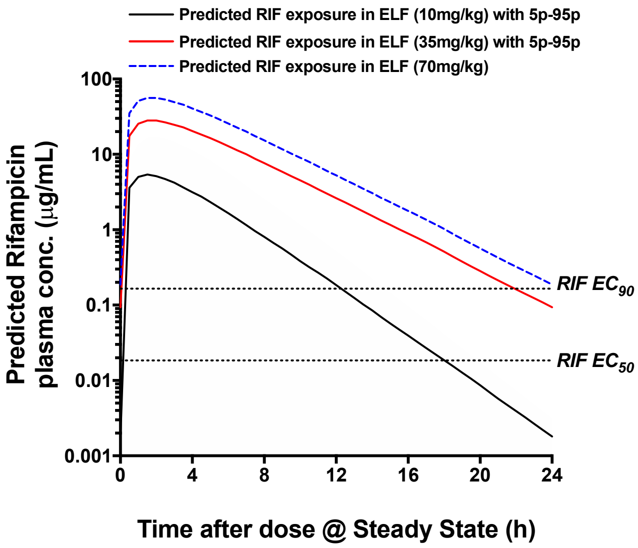

Figure 2. RIF predicted plasma exposure at different doses overlaid with RIF EC_{50} and EC_{90}.
